# Cluster Persistence for Weighted Graphs

**DOI:** 10.3390/e25121587

**Published:** 2023-11-26

**Authors:** Omer Bobrowski, Primoz Skraba

**Affiliations:** 1 School of Mathematical Sciences, Queen Mary University of London, London E1 4NS, UK; 2 Viterbi Faculty of Electrical and Computer Engineering, Technion, Haifa 3200003, Israel; 3 Department for Artificial Intelligence, Jozef Stefan Institute, 1000 Ljubljana, Slovenia

**Keywords:** topological data analysis, universality, clustering

## Abstract

Persistent homology is a natural tool for probing the topological characteristics of weighted graphs, essentially focusing on their 0-dimensional homology. While this area has been thoroughly studied, we present a new approach to constructing a filtration for cluster analysis via persistent homology. The key advantages of the new filtration is that (a) it provides richer signatures for connected components by introducing non-trivial birth times, and (b) it is robust to outliers. The key idea is that nodes are ignored until they belong to sufficiently large clusters. We demonstrate the computational efficiency of our filtration, its practical effectiveness, and explore into its properties when applied to random graphs.

## 1. Introduction

**Figure 1:**
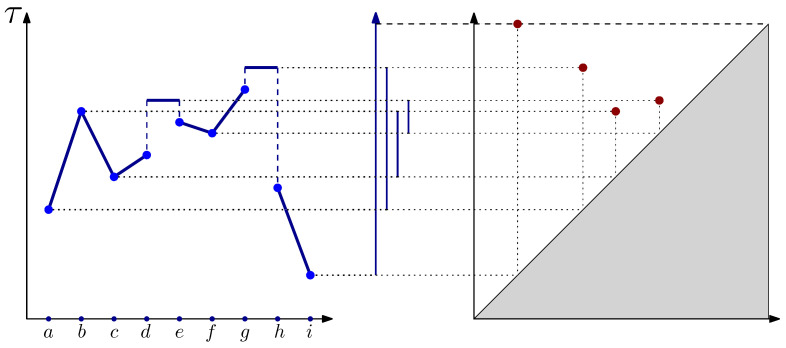
An example of graph filtration on a line graph. The filtration values of the vertices are given by τ (the *y*-axis). The filtration value of each edge is taken as the highest value between its plotted endpoints. The bars in the middle represent the tracking of the components. The vertices that are local minima, i.e., *a*, *c*, *f*, and *i*, generate new components; so, τ(a), τ(c), τ(f), and τ(i) correspond to birth times. The first merge occurs at τ(b)=τ((a,b))=τ((b,c)), merging $\left\{a\right\}$ with $\left\{c,d\right\}$. In this case, we declare the latter as dead since τ(a)<τ(c). Next, at τ((d,e)), the components $\left\{a,b,c,d\right\}$ and $\left\{e,f\right\}$ are merged and the latter dies. Finally, at τ((g,h)), the components $\left\{a,b,c,d,e,f,g\right\}$ and $\left\{h,i\right\}$ are merged and the former dies. The component containing *i* has the earliest birth time and is thus declared to be infinite.

The **clustering** of data is a fundamental task in unsupervised machine learning and exploratory data analysis. It has been the subject of countless studies over the last 50 years, with many definitions and algorithms being proposed, e.g., [1,2]. **Persistent homology** [3,4] is a powerful topological tool that provides multi-scale structural information about data and networks [5,6,7,8]. Given an increasing sequence of spaces (filtration), persistent homology tracks the formation of connected components (0-dimensional cycles), holes (1-dimensional cycles), cavities (2-dimensional cycles), and their higher-dimensional extensions. The information encoded in persistent homology is often represented by a *persistence diagram*, which is a collection of points in $R^{2}$ representing the birth and death of homology classes and providing an intuitive numerical representation for topological information (see Figure 1).

The connection between clustering and 0-dimensional persistent homology is well established in various different scenarios. Specifically, the relationship with functoriality [9,10] has been investigated, as well as how to combine persistence with density-based methods [11,12]. An important motivating factor for connecting these methods is *stability*. Namely, given small perturbations in the input data, persistent homology can provide guarantees on the number of the output clusters. One important drawback of this topological approach is that statistical tests for persistent homology and clustering based on persistence remain lacking.

Recently, for persistent homology in dimensions 1 and above (i.e., excluding connected components), persistent homology based on distance filtration has been experimentally shown to exhibit strong universal behavior [13]. Suppose we are given as the input a point cloud generated by some unknown distribution. If we compute the distance-based persistent homology, under an appropriate transformation, the distribution of persistence values has been shown to be independent of the original point cloud distribution. This phenomenon has since been used to develop a statistical test to detect statistically significant homology classes. A key point in [13] is that in order to obtain such universal behavior, the measure of persistence must be given by the value of death/birth, which makes the measure of persistence scale-invariant.

However, in distance-based filtration, 0-dimensional persistent homology (tracking clusters) does not fit into this universality framework as the birth time of every 0-dimensional homology class is set to 0. To address this issue and enable the study of universality in the context of clustering, we introduce a new filtration approach, which we call *k*-cluster filtration. This is a novel non-local construction, where vertices only become ‘alive’ once they belong to a sufficiently large cluster. In other words, while traditional persistent homology considers every vertex as an individual cluster and tracks its evolution, *k*-cluster filtration only considers components with *k* or more vertices as ‘meaningful’ clusters.

We note that while the motivation for this new filtration method is to study distance-based filtrations, *k*-cluster filtration can be constructed over any weighted graph. It generally provides two key advantages over traditional filtration. Firstly, it results in ‘richer’ persistence diagrams, in the sense that components have non-trivial birth times. This improves our ability to compare between different features within the same diagram or across different diagrams. Secondly, *k*-cluster filtration provides a more ‘focused’ view of connected components by discarding those that are considered small (determined by application). In particular, it allows us to remove outliers from persistence diagrams.

This paper is organized as follows: Section 2 provides essential background information about persistent homology; Section 3 introduces *k*-cluster filtration and presents some preliminary properties; Section 4 provides an algorithm for computing the filtration function and its corresponding persistence diagram in a single pass; Section 5 demonstrates some experimental results comparing our clustering method to some other approaches; and finally, Section 6 discusses some probabilistic aspects of this filtration method by comparing it to known properties of random graphs and simplicial complexes.

## 2. Graph Filtration and Persistent Homology

We first introduce the required topological notions. As we focus on the special case of graphs and connected components (0-dimensional homology), we restrict our definitions to this case. For the general definition of homology, we refer the reader to [14,15].

Let G=(V,E) be an undirected graph. Our main object of study is *graph filtration*, i.e., an increasing sequence of graphs. This can be constructed by defining a function τ:(V∪E)→[0,∞), with the restriction that if e=(u,v)∈E, then τ(e)≥max(τ(u),τ(v)). This restriction ensures that the sublevel sets of τ define a subgraph. The filtration ${\left\{G_{t}\right\}}_{t\geq 0}$ is then defined as

$$G_{t}=\left\{\sigma \in V\cup E:\tau (\sigma )\leq t\right\}.$$

As we increase *t* from 0 to *∞*, we can track the connected components of $G_{t}$ as they appear and merge, which are referred to as *births* and *deaths*, respectively. When two components merge, we use the ‘elder rule’ to determine that the component that was born last is the one that dies. Note that at least one component has an infinite death time in any graph filtration. We refer the reader to [3] for an in-depth introduction to persistent homology.

These birth–death events can be tracked using an algebraic object called the 0-dimensional persistent homology group. Its most common visualization is a *persistence diagram*, which is a collection of points in $R^{2+}$ where each point corresponds to a single connected component. The coordinates of a point encode its information, with the *x*-coordinate representing the birth time and the *y*-coordinate representing the death time. An example of a function on a line graph is shown in Figure 1. Note that one component is infinite, which is denoted by the dashed line at the top of the diagram.

In a more general context, given the filtration of higher-dimensional objects (e.g., simplicial complexes), we can also study *k*-dimensional persistent homology. This object tracks the formation of *k*-dimensional cycles (various types of holes) and its definition is a natural extension of the 0-dimensional persistent homology we study here. However, this is beyond the scope of this paper and we refer the reader to [3] for more information.

## 3. k-Cluster Filtration

Let G=(V,E,W) be an undirected weighted graph. When computing 0-dimensional persistent homology, the filtration values are commonly taken to be τ(v)=0 for all v∈V and τ(e)=W(e) for all e∈E. We denote this filtration by $G_{t}^{*}$. In other words, we assume all vertices are present at time zero and that edges are gradually added according to the weight function *W*. This has been common practice in almost all studies in the TDA literature, particularly in geometric settings where *W* represents the distance between points, i.e., geometric graphs, which are the skeleton of two common constructions: the Čech and Vietoris–Rips complexes [3]. While in many models, this choice of τ seems reasonable, it has two significant drawbacks:

- The produced persistence diagrams are *degenerate* as the birth time of every 0-cycle is t=0, which significantly reduces the amount of information we can extract from the diagrams;
- The generated persistence diagrams are *superfluous* in the sense that they contain a point for each vertex *V*, while obviously not all vertices contribute significant structural information.

In this paper, we propose a modification to standard graph filtration that will resolve both of these issues and lead to more concise and informative persistence diagrams.

We will first define the filtration values for the vertices. For every vertex and value t>0, we define $N_{t}\left(v\right)$ to be the number of vertices in the connected component of $G_{t}^{*}$ that contains *v*.Fix k≥1, and define

(1) $$\tau_{k}\left(v\right):=\inf\left\{t:N_{t}\left(v\right)\geq k\right\}.$$

The edge values are then defined as

(2) $$\tau_{k}\left(\left(u,v\right)\right)=\max\left(\tau_{k}\left(u\right),\tau_{k}\left(v\right),W\left(\left(u,v\right)\right)\right).$$

Denoting the corresponding filtration by $G_{t}^{\left(k\right)}$, note that $G_{t}^{\left(1\right)}\equiv G_{t}^{*}$. In other words, compared to $G_{t}^{*}$, in $G_{t}^{\left(k\right)}$, we delay the appearance of the vertices until the first time each vertex is contained in a component with at least *k* vertices (and adjust the edge appearance to be compatible). Effectively, the assignment of the new filtration values to the vertices introduces three changes to persistence diagrams:

1. All points that are linked to components smaller than *k* are removed;
2. Each birth time corresponds to an edge merging two components $C_{1},C_{2}$ in $G_{t}^{*}$, such that $\left|C_{1}\right|,\;|C_{2}|\;<k$ and $\left|C_{1}\right|\;+\;|C_{2}|\;\geq k$;
3. Each death time corresponds to an edge merging two components with at least *k* vertices each.

We call this filtration approach ‘*k*-cluster filtration’ to represent the fact that it tracks the formation and merging of clusters of at least *k*. The parameter *k* determines what we consider to be a sufficiently meaningful cluster. In $G_{t}^{*}$, every vertex is considered to be a cluster but, statistically speaking, this is overkill. The chosen value of *k* should depend on the application, as well as the sample size.

We conclude this section by showing that *k*-cluster filtration decreases (in a set sense) as we increase *k*. This could be useful, for example, in the context of multi-parameter persistence, which we briefly mention later but leave for future work.

**Lemma 1.**  *Filtration $G_{t}^{\left(k\right)}$ decreases in k or equivalently:*

$$\tau_{k-1}\left(x\right)\leq \tau_{k}\left(x\right),\;\forall x\in V\cup E.$$

**Proof.**  For any vertex v∈V, if $|N_{t}\left(v\right)|\geq k$, then $|N_{t}\left(v\right)|\geq k-1$. From (1), we therefore have that $\tau_{k-1}\left(v\right)\leq \tau_{k}\left(v\right)$. Using (2), we have $\tau_{k-1}\left(e\right)\leq \tau_{k}\left(e\right)$ for all e∈E.    □

## 4. Algorithm

In this section, we describe an efficient one-pass algorithm for computing the filtration function and persistence diagram at the same time. The time complexity of the algorithm is O(|E|×α(|V|)), where α(·) is the inverse Ackermann function [16]. This is the same complexity as when computing 0-dimensional persistence diagrams if we were given the filtration function as input.

We begin with the (standard) terminology and data structures. For simplicity, we assume that the weights of the edges are unique and that the vertices have a lexicographical order. We first define the filtration function, which determines the total ordering of the vertices. Initially, undefined filtration function values are assumed to be *∞*. If the function value is the same or undefined for two vertices, the order is determined via lexicographical ordering. It is then straightforward to check this is a total ordering.

**Remark 1.**  *In the case of a total ordering, we can choose a representative 0-dimensional persistent homology class. Notably, in total ordering, a unique vertex is the earliest generator for a homology class (i.e., cluster), which we denote as a canonical representative of the persistent component.*

To track components as we proceed incrementally through the filtration approach, we use the union-find data structure, which supports two operations:

- ROOT(v) returns the canonical representative for the connected component containing *v*;
- MERGE(u,v) merges the connected components containing *u* and *v* into one component, including updating the root.

We augment the data structure by keeping track of two additional records:

- SIZE(v) returns the size of the connected component containing *v*;
- COMPONENT(v) returns the list of vertices in the same component as *v*.

To track the size of the component, we store the size at the root (i.e., the canonical representative) of each component, updating it each time a merge occurs. To access a connected component, recall that union-find data structures can be implemented as rooted trees. For each vertex, we store a list of children in the tree. To recover the list of vertices in the component, we perform a depth-first search of the tree, starting from the root (although any other traversal method could be used). All update operations have O(1) cost (cf., [16]).

Note that when k=1, the filtration value of each vertex is 0; so, the problem reduces to finding the minimum spanning tree of a weighted graph. Hence, we assume that k>1. Initially, we set the filtration functions τ(v)=0 for all vertices and τ(e)=W(e) for all edges and assume that the edges are sorted by increasing weight. Note that if this is not the case, this step will become a bottleneck, with a cost of O(|E|log|E|). Thus, we begin with a forest where each component is a single vertex, i.e., all components are initially born at 0.

We proceed as in the case of standard 0-dimensional persistence, by adding edges incrementally. As all components are present at time 0, we are only concerned with merges. The problem is reduced to updating birth times as we proceed by keeping track of ’active’ components, i.e., those larger than *k*. We omit points in persistence diagrams that are on the diagonal (death = birth), although these may be included with some additional bookkeeping.

Assume we are adding the edge e=(u,v). If *e* is internal to a connected component (i.e., ROOT(u)=ROOT(v)), then it does not affect the 0-persistence. Otherwise, it connects the two components denoted as $C_{u},C_{v}$. There are a few cases to consider:

1. $|C_{u}\cup C_{v}|\;<k$: The merged component is too small to affect the persistence diagram, so we only perform a merge of the components;
2. $|C_{u}\cup C_{v}|\;\geq k$ and $|C_{u}|\;<k$: In this case, $C_{u}$ becomes active. Thus, we merge the components and update the value of τ for all vertices in $C_{u}$.
  $$\tau \left(x\right)\leftarrow W\left(e\right)\;\;\forall x\in C_{u}$$
  is performed. We take similar action if $|C_{v}|\;<k$ (or if both are less than *k*);
3. $|C_{u}\left|,\right|C_{v}|\;\geq k$: Both components are already active and so a new point (birth, death) is added to the persistence diagram using
  $$\begin{array}{cc} \mathrm{birth} & =\max\left\{\tau (\mathrm{ROOT}(u)),\tau (\mathrm{ROOT}(v))\right\},\\ \mathrm{death} & =W(e). \end{array}$$
  The components are again merged. We note that for any *v*,
  $$\tau \left(\mathrm{ROOT}\left(v\right)\right)=\min_{x\in C_{v}}\tau \left(x\right).$$

The full procedure is given in Algorithm 1. Note that we only compute the filtration of the vertices as the correct edge values can then be computed using Equation (2).

**Proof of Correctness.**  We first argue that the function τ is correctly computed. This follows directly from the fact that the algorithm explicitly tests that the components contain at least *k* vertices. The fact that the corresponding persistence diagram is correctly computed is a consequence of the following result.    □

**Lemma 2.**  *The minimum spanning tree for k=1 is the minimum spanning tree for any k.*

**Proof.**  The key observation is that until a component contains *k* vertices, any spanning tree is a minimum spanning tree as all edges are assigned the value of when the component becomes active. The values of the remaining edges do not change and so remain in the MST.    □

The equivalence of the MST and persistence diagram [17] then implies the correctness of the algorithm.

**Proof of Running Time.**  The analysis of the merging is covered verbatim in the standard analysis of union-find data structures [16]. As described above, updating the sizes of the components and the lists of children in the merges are O(1) operations. All that remains to prove is the cost of updating the function τ. We observe that each vertex is only updated once. This has a total cost of O(|V|), while the edges can be updated at a cost of O(1) each. However, if we only want to obtain a persistence diagram (and not the actual graph filtration), we do not need to update the edges since we perform a single pass. Therefore, the overall running time is O(|E| ×α(|V)).    □

**Extracting the Clusters.**  To obtain clusters, we can use the algorithm in [12]. This algorithm extracts the *ℓ*-most persistent clusters by only performing merges when the resulting persistence is less than a given threshold. This threshold can be chosen such that there are only *ℓ* points above the threshold in the diagram. Finally, we note that cluster extraction can be performed on an MST rather than a full graph.

**Algorithm 1** The one-pass algorithm
1:

G=(V,E,W)

2:

τ:V→[0,∞)

3:Initialize union-find data structure: ROOT(v)=v for all v∈V4:

Dgm, MST=∅

5:**for** e=(u,v)∈E **do**6:    **if** ROOT(u)≠ROOT(v) **then**7:        MST←MST∪e8:        **if** SIZE(u)+SIZE(v)>k **then**9:           **if** SIZE(u)<k **then**10:               **for** x∈COMPONENT(u) **do**11:                   τ(x)←W(e)12:               **end for**13:           **end if**14:           **if** SIZE(v)<k **then**15:               **for** x∈COMPONENT(v) **do**16:                   τ(x)←W(e)17:               **end for**18:           **end if**19:           **if** SIZE(u),SIZE(v)≥k **then**20:               birth=max{τ(ROOT(u)),τ(ROOT(v))}21:               death=W(e)22:               Dgm←Dgm∪(birth,death)23:           **end if**24:        **end if**25:        MERGE(u,v)26:    **end if**27:
**end for**
28:**return** Dgm,MST,τ


## 5. Experiments and Applications

In this section, we probe the behavior of *k*-cluster persistence through a sequence of experiments. Using several synthetic datasets, we study the dependence on the parameter *k* and experimentally show that this filtration approach follows the universality laws discovered in [13]. Next, we demonstrate how *k*-cluster persistence can be used for clustering by taking advantage of universality to automatically determine the number of ’statistically significant’ clusters. Finally, we show how our new clustering method works in more exotic spaces, specifically tree structures. The results presented here are a preliminary investigation into this construction and we expect to continue with more applications in future work.

**Remark 2.**  *Note that in all persistence diagrams presented in the figures, the axes are modified to birth vs. death/birth (rather than birth vs. death).*

### 5.1. Simulated Point Clouds

**Figure 2:**
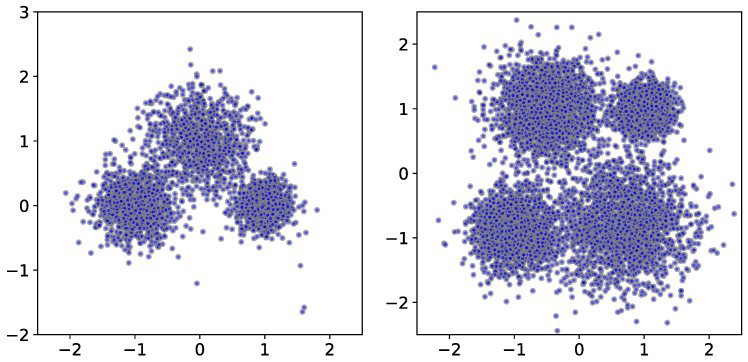
Two examples of point clouds consisting of IID sampling from a mixture of three and four Gaussian functions, with 1000 and 2000 points, respectively.

**Figure 3:**
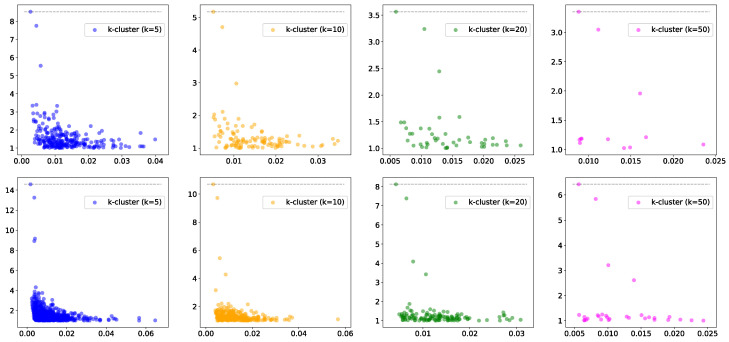
The persistence diagrams, with death/birth on the *y*-axis and different values of *k* for the points sampled from the two mixtures of Gaussian functions (**top** row: 3 groups; **bottom** row: 4 groups). Note that the number of outstanding features in the diagrams correspond to the number of clusters in the data.

We start by generating point clouds from a mixture of Gaussians, resulting in several clusters of points (Figure 2). We first show the effect of the parameter *k* on the filtration function and the corresponding persistence diagrams. For the two point clouds in Figure 2, we show the resulting persistence diagrams for the *k*-cluster filtration functions in Figure 3. Notice that the correct number of persistent clusters is evident, especially for k=10, 20, and 50. An important phenomenon that is evident in the figures is that higher values of *k* filter out more of the ‘noise’.

To place the behavior of the persistence diagrams into further context, we compare the *k*-cluster filtration method to a related construction from the applied topology literature, which has been suggested for dealing with outliers in clustering (and higher homological dimensions): *k*-degree Vietoris–Rips filtration [18]. Given a weighted graph G=(V,E,W), we define the *k*-degree filtration function $\delta_{k}:\left(V\cup E\right)\to \left[0,\infty \right)$ as follows: for every vertex v∈V, we take $\delta_{k}\left(v\right)$ to be its *k*-nearest neighbor distance. The values of the edges are then determined as in (2). The *k*-degree filtration approach has been used in the context of multi-parameter persistence, with bifiltration induced by decreasing *k* and increasing the edge weight (commonly, Euclidean distance). In this paper, we do not explore multi-parameter settings. Rather, we focus the properties of persistence diagrams for fixed values of *k*. We make two observations before investigating the differences:

1. The *k*-degree filtration function is determined completely by the local neighborhood of a vertex, i.e., its immediate neighbors in the graph. The same is not true for *k*-cluster filtration;
2. For a fixed value of *k*, we have $\tau_{k}\left(v\right)\leq \delta_{k-1}\left(v\right)$ for all v∈V. In other words, the value of the *k*-cluster function is less than or equal to the value of the (k−1)-degree function. This follows from the fact that if a vertex has k−1 neighbors, then it is part of a cluster with at least *k* vertices.

**Figure 4:**
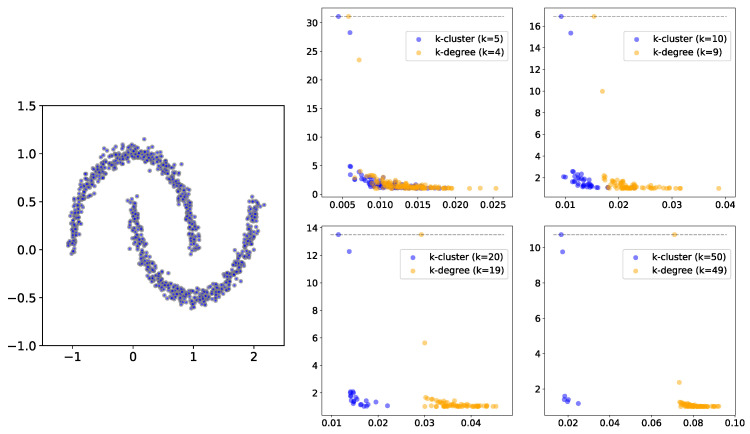
A comparison of *k*-cluster and *k*-degree filtrations for the two moons dataset. On the right, we have the persistence diagrams for different values of *k*.

**Figure 5:**
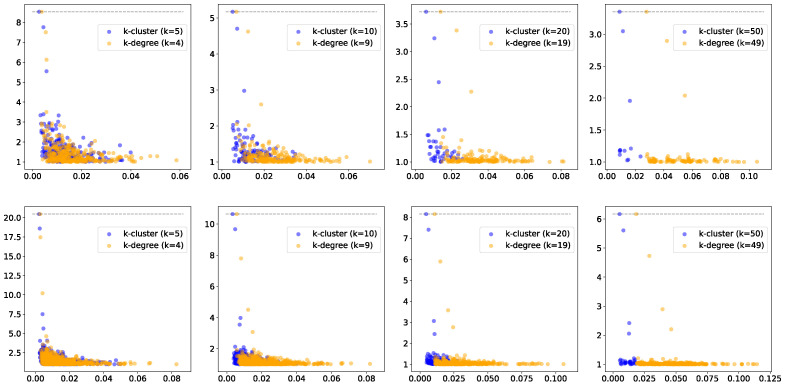
The persistence diagrams for each point cloud, with *k*-degree filtration presented in yellow and *k*-cluster filtration presented in blue for k=5,10,20, and 50 (**top row**: 3 clusters; **bottom row**: 4 clusters).

**Figure 6:**
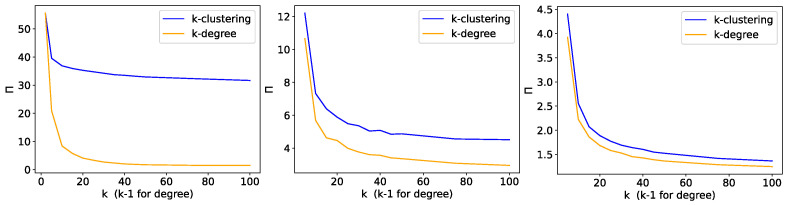
The effect on the second most persistent cluster for different values of *k*. In the left and center panels, this corresponds to true clusters (**left**: two moons; **center**: a mixture of 3 Gaussians). The right panel presents the results for uniformly random points. Here, the noise cluster drops nearly as quickly in both cases.

In Figure 4, we show the persistence diagrams (i.e., birth vs. death/birth) for two non-convex clusters for both the *k*-degree and *k*-cluster filtration methods, using different values of *k*. In this example, especially for larger *k* values, the persistent clusters are much more prominent in *k*-cluster filtration compared to *k*-degree filtration. This may be explained by the fact that a much larger radius is needed to obtain the required number of neighbors. In Figure 5, we show the same comparison for persistence diagrams for 3 and 4 groups, where the difference between the two methods is less clear. However, Figure 6 highlights an additional difference between the behaviors of the two filtration approaches. In this figure, we compare persistence (death/birth) for the second most persistent cluster, using a wide range of *k* values. In the left and center plots, the second most persistent clusters correspond to true clusters in the data. We observe that the persistence value decays much more slowly for *k*-cluster filtration, i.e., the true cluster remains more persistent for increasing values of *k*. The plot on the right presents the same comparison but for uniformly distributed random points. In this case, the second most persistent cluster is construction noise, i.e., not a real cluster in the data. Although *k*-cluster filtration still decays more slowly, it is at a comparable rate to that of *k*-filtration. Hence, we can conclude that persistent clusters show more stable behavior over ranges of *k* for *k*-cluster filtration compared to *k*-degree filtration.

### 5.2. Universality

In [13], we published a comprehensive experimental work showing that the distribution of persistence values is universal. We consider persistence diagrams as finite collections of points in $R^{2}$: $\mathrm{dgm}=\left\{\left(b_{1},d_{1}\right),\ldots ,\left(b_{M},d_{M}\right)\right\}$. For each point $p_{j}\;=\;\left(b_{j},d_{j}\right)$, we consider the multiplicative persistence value $\pi \left(p_{j}\right)=d_{j}/b_{j}$. Our goal is to study the distributions of π-values across entire diagrams.

Our results in [13] are divided into two main parts. Given a point cloud of size *n*, we compute the persistence diagrams for Čech and Vietoris–Rips filtrations. In *weak universality*, we consider the empirical measure

$$\Pi_{n}:=\frac{1}{|{\mathrm{dgm}}_{i}|}\sum_{p\in {\mathrm{dgm}}_{i}}\delta_{\pi (p)},$$

and we conjecture that for IID samples, we have

$$\lim_{n\to \infty }\Pi_{n}=\Pi_{d,T,i}^{*},$$

where *d* is the dimension of the point cloud, *i* is the degree of homology, and T is the filtration type, i.e., Čech or Vietoris–Rips. In other words, the limiting distributions for the π-values depend on d,i,T but are independent of the probability distributions generating the point clouds.

In *strong universality*, we present a much more powerful and surprising conjecture. Here, we define ℓ(p):=Aloglog(π(p))+B (the values of *A* and *B* are specified in [13]) and the empirical measure

$$L_{n}:=\frac{1}{|{\mathrm{dgm}}_{i}|}\sum_{p\in {\mathrm{dgm}}_{i}}\delta_{\ell (p)}.$$

Our conjecture is that for wide class of random point clouds (including non-IID and real data), we have

$$\lim_{n\to \infty }L=L^{*},$$

where $L^{*}$ is a unique universal limit. Furthermore, we conjecture that $L^{*}$ might be the left-skewed Gumbel distribution.

**Figure 7:**
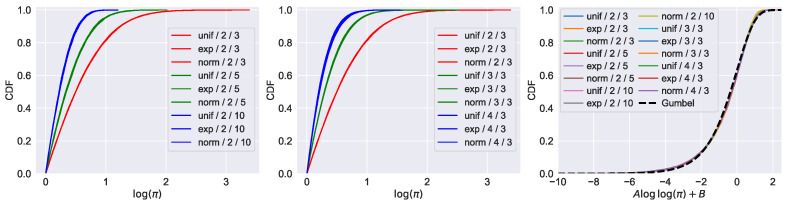
Universal distribution for *k*-cluster persistence. The labels in the legend are structured as distribution/*d*/*k*, where *d* is the dimension of the point cloud and *k* is the cluster size. The distributions taken are uniform in unit boxes, exponential, and normal. The first two plots show that weak universality holds and that the limit depends on d,k, but not on the distribution. The rightmost plot demonstrates that strong universality holds under proper normalization. We also include the left-skewed Gumbel distribution (dashed line) for comparison.

Originally, the results in [13] were irrelevant for the 0-th persistence diagrams of random point clouds as the birth times were all zero. However, once we replace standard filtration with *k*-cluster filtration, we obtain new persistence diagrams with non-trivial birth times that we can study. In Figure 7, we demonstrate both weak and strong universality properties for *k*-cluster persistent homology. We generated IID point clouds across different dimensions and with different distributions (uniform in a box, exponential, normal). The results show that both weak and strong universality hold in these cases as well. We note that for weak universality, the limiting distribution depends on both *d* (the dimension of the point cloud) and *k* (the minimum cluster size).

### 5.3. Clustering

As mentioned in the introduction, a key motivation for this work was to apply *k*-cluster filtration to clustering. To obtain clustering from a 0-dimensional persistence diagram, we use the algorithm proposed in [12]. Roughly speaking, given a threshold α, the algorithm extracts all clusters that are at least α-persistent. We note that the original measure for persistence in [12] was given by d−b; however, the change to use d/b in the algorithm is trivial.

**Statistical Testing.**  An important consequence of the universality results presented in Section 5.2 is that the limiting distribution (after normalization) appears to be a known distribution, i.e., the left-skewed Gumbel. We can thus perform statistical testing on the number of clusters, as in [13]. The null hypothesis denoted by $H_{0}^{\left(i\right)}$ is that the *i*-th most persistent cluster is due to noise. Assuming that the universality conjectures hold, the null hypothesis is given in terms of the *ℓ*-values as

$$H_{0}^{\left(i\right)}\;:\;\ell \left(p_{i}\right)∼\mathrm{LGumbel}.$$

where $p_{i}$ represents the *i*-th most persistent cluster in terms of death/birth. The corresponding *p*-value is given by

$$p{-\mathrm{value}}_{i}=P\left(\ell \left(p_{i}\right)\geq x\;|\;H_{0}^{\left(i\right)}\right)=e^{-e^{x}}.$$

Note that since we are testing sorted values, we must use multiple hypothesis testing corrections. In the experiments we describe below, we use the Bonferroni correction.

**Figure 8:**
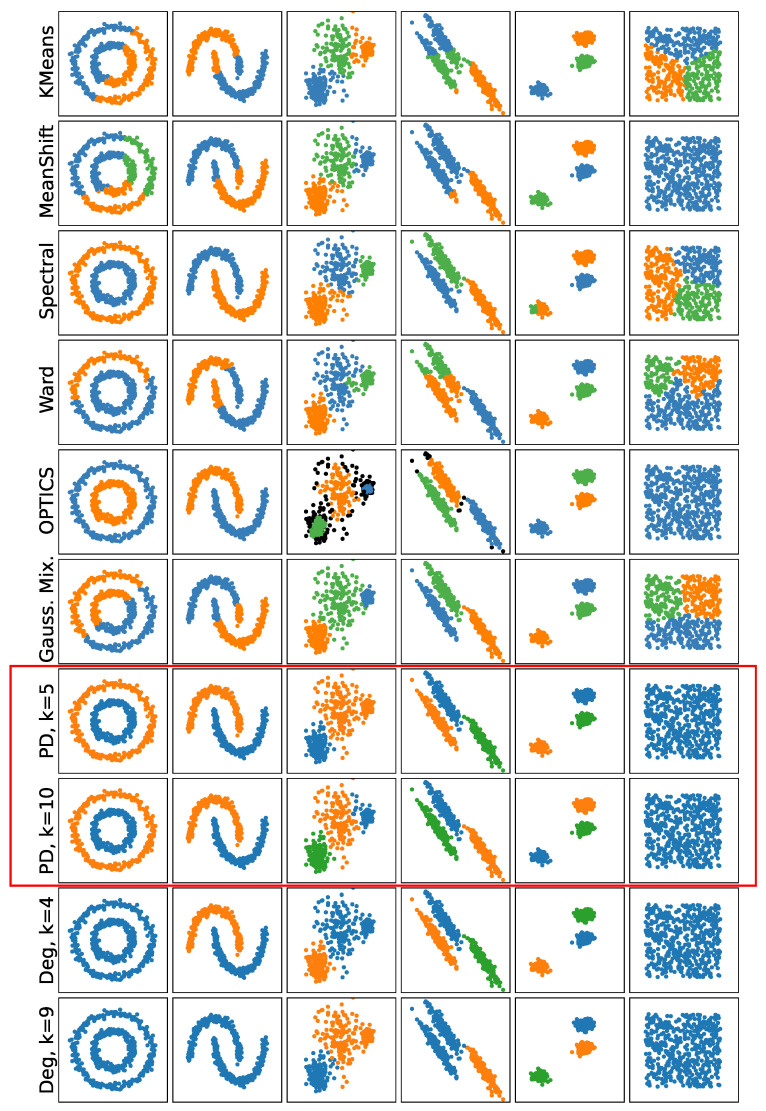
A comparison of standard clustering examples using different clustering approaches. In the case of *k*-cluster filtration (PD) and *k*-degree filtration (Deg), the numbers of clusters are chosen using statistical significance testing.

In Figure 8, we compare *k*-cluster filtration and *k*-degree filtration using persistence-based clustering from [12] and other common algorithms for clustering. For the other approaches, we use the standard implementations found in [19], which have associated techniques for choosing the number of clusters. In the cases of *k*-cluster filtration and *k*-degree filtration, the numbers of clusters are chosen using the statistical testing described above. Note that since the numbers of points in the standard examples are quite small, we limit *k* to 5 and 10. The best result is for *k*-cluster filtration with k=10 (k=5 fails to identify one of the clusters in the third example). On the other hand, *k*-degree filtration performs well but the additional ‘noise’ points in the diagram mean that some clusters are not identified as significant.

**Figure 9:**
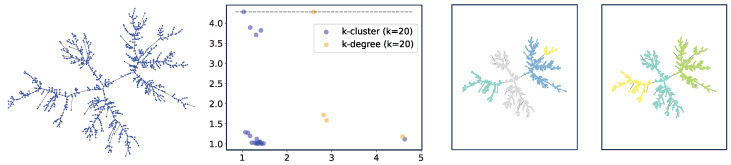
Clustering on a uniform random tree. The threshold gives 4 clusters for *k*-cluster filtration but only 3 for (metric) *k*-degree filtration.

**Clustering on Trees.**  As a second example, we describe clustering on weighted trees. We generate a uniform random tree on *n* vertices and assign uniformly distributed random weights on the edges (between 0 and 1). We show an example in Figure 9. The method seems to capture a certain structure of the tree, although we leave further investigation of this structure as future work.

Note that in the tree case, it is often impossible to use *k*-degree filtration as the trees have vertices with degrees that are smaller than *k*, which will never be included in filtration, whereas for *k*-cluster filtration, all nodes are included as long as the underlying graph is connected (or all components have at least *k* vertices). We note that it is possible to use alternative definitions for *k*-degree filtration by embedding the trees into metric spaces (i.e., using graph metrics induced by the weights). However, this is similar to studying complete graphs induced by the metrics, which is somewhat different from studying the graphs directly. We demonstrate this method in the rightmost plot of Figure 9.

## 6. Probabilistic Analysis

In this section, we revisit some fundamental results for random graphs and random simplicial complexes and show that analogous statements hold for our new *k*-cluster filtration approach. We provide the main statements, while the proofs are available in the Appendix A, Appendix B and Appendix C. As real data are random, we view these statements (and their future expansions) as an integral part of the analysis of *k*-cluster persistence, within the context of topological data analysis.

### 6.1. Connectivity

We consider two models here. In the G(n,p) random graph, we have *n* vertices and each edge is placed independently with probability *p*. In the G(n,r) random geometric graph, we take a homogeneous Poisson process $P_{n}$ on the *d*-dimensional flat torus, with rate *n*. Edges are then placed between vertices that are less than *r* apart. In both models, connectivity results are tied to the expected degree. For the G(n,p) model, we define Λ=np, while for the G(n,r) model, we take $\Lambda =n\omega_{d}r^{d}$. Then, in [20,21], the following was proved.

**Theorem 1.**  *Let $G_{n}$ be either G(n,p) or G(n,r). Then*

$$\lim_{n\to \infty }P\left(G_{n}\;\mathrm{is}\;\mathrm{connected}\right)=\left\{\begin{array}{cc} 1 & \Lambda =\log n+w(n),\\ 0 & \Lambda =\log n-w(n). \end{array}\right.$$

A key element in proving connectivity (for either model) is to show that around Λ=logn, the random graph consists of a single giant component, a few isolated vertices, and nothing else. Thus, connectivity is achieved when the last isolated vertices become connected.

Our goal in this section is to analyze connectivity in the G(n,p) and G(n,r) models using our new *k*-cluster filtration method. Note that for a fixed value of *n*, we can view both models as filtrations over the complete graphs. For the G(n,p) model, the weights of the edges are independent random variables that are uniformly distributed in [0,1]. For the G(n,r) model, the weight of an edge is given by the distance between the corresponding points in the torus. We define $G^{\left(k\right)}\left(n,p\right)$ and $G^{\left(k\right)}\left(n,r\right)$ as the random filtrations generated by changing the filtration function to $\tau_{k}$. Our goal here is to explore the phase transitions for *k*-cluster connectivity. As opposed to connectivity in the original random graphs, these results differ between the models.

**Theorem 2.**  *For the $G^{\left(k\right)}\left(n,p\right)$ filtered graph, we have*

$$\lim_{n\to \infty }P\left(G^{\left(k\right)}\left(n,p\right)\;\mathrm{is}\;\mathrm{connected}\right)=\left\{\begin{array}{cc} 1 & \Lambda =\frac{1}{k}\left(\log n+\left(k-1\right)\log\log n\right)+w\left(n\right),\\ 0 & \Lambda =\frac{1}{k}\left(\log n+\left(k-1\right)\log\log n\right)-w\left(n\right), \end{array}\right.$$

*for any w(n)=o(loglogn), such that w(n)→∞.*

For the $G^{\left(k\right)}\left(n,r\right)$ model, proving the connectivity is a much more challenging task and beyond the scope of this paper. However, the following statement is relatively straightforward to prove.

**Proposition 1.**  *Let $N_{k}=N_{k}\left(n,r\right)$ be the number of connected components of size k in G(n,r). Then,*

$$\lim_{n\to \infty }P\left(N_{k}=0\right)=\left\{\begin{array}{cc} 1 & \Lambda =\log n-(d-1)(k-1)\log\log n+w(n),\\ 0 & \Lambda =\log n-(d-1)(k-1)\log\log n-w(n). \end{array}\right.$$

*for any w(n)=o(loglogn), such that w(n)→∞.*

From this lemma, we conclude that when Λ=logn−(d−1)(k−1)loglogn−w(n), the graph G(n,r) has components of size *k*, which implies that $G^{\left(k\right)}\left(n,r\right)$ is not connected. On the other hand, when Λ=logn−(d−1)(k−1)loglogn+w(n), we have $N_{j}=0$ for all fixed j≥k, which indicates that $G^{\left(k\right)}\left(n,r\right)$ should be connected. This leads to the following conjecture.

**Conjecture 3.**  *For the $G^{\left(k\right)}\left(n,r\right)$ filtered graph, we have*

$$\lim_{n\to \infty }P\left(G^{\left(k\right)}\left(n,r\right)\;\mathrm{is}\;\mathrm{connected}\right)=\left\{\begin{array}{cc} 1 & \Lambda =\log n-(d-1)(k-1)\log\log n+w(n),\\ 0 & \Lambda =\log n-(d-1)(k-1)\log\log n-w(n). \end{array}\right.$$

Note that both phase transitions occur before those for the original graph models. This is due to the fact that for k>1, *k*-cluster filtration does not allow any isolated vertices. Also note that when taking k=1, both results coincide with Theorem 1.

### 6.2. Limiting Persistence Diagrams

In [22], it is shown that for stationary point processes, persistence diagrams have non-random limits (in the vague convergences of measures). A similar statement holds for *k*-cluster persistence diagrams.

Let ${\mathrm{Dgm}}^{\left(k\right)}\left(P\right)$ be the *k*-cluster persistence diagram for point cloud P. We define the discrete measure on $R^{2}$ as

$$\xi^{\left(k\right)}\left(P\right):=\sum_{\left(b,d\right)\in {\mathrm{Dgm}}^{\left(k\right)}\left(P\right)}\delta_{\left(b,d\right)}.$$

Let $Q_{L}={\left[-L/2,L/2\right]}^{d}$. The following is an analog of Theorem 1.5 in [22].

**Theorem 4.**  *Assume that P is a stationary point process in $R^{d}$, with all finite moments. For any k, there exists a deterministic measure $\mu_{k}$, such that*

$$\lim_{L\to \infty }\frac{1}{L^{d}}E\left\{\xi^{\left(k\right)}\left(P\cap Q_{L}\right)\right\}=\mu_{k},$$

*where the limit is in the sense of vague convergence. Furthermore, if P is ergodic, then almost surely*

$$\lim_{L\to \infty }\frac{1}{L^{d}}\xi^{\left(k\right)}\left(P\cap Q_{L}\right)=\mu_{k}.$$

### 6.3. Maximal Cycles

In [23], the largest cycles in persistence diagrams are studied. In particular, the behavior of the largest π-value: π(p)=d/b arising from a homogeneous Poisson process $P_{n}$ is studied. Let $\Pi_{i,\max}$ be the largest π-value in the *i*-th persistent homology. The main result in [23] then states that, with high probability,

$$A_{i}\Delta_{i}\left(n\right)\leq \Pi_{i,\max}\leq B_{i}\Delta_{i}\left(n\right),$$

where $A_{i},B_{i}>0$ are constants, and

$$\Delta_{i}\left(n\right)={\left(\frac{\log n}{\log\log n}\right)}^{1/i}.$$

For *k*-cluster persistence, we show that the largest π-value has a completely different scaling.

**Theorem 5.**  *Let $P_{n}$ be a homogeneous Poisson process in the flat torus, with rate n. Let $\Pi_{\max}^{\left(k\right)}$ denote the maximum π-value in the k-cluster persistence diagram (excluding the infinite cluster). Then, for every ϵ>0, we have*

$$\lim_{n\to \infty }P\left(n^{\frac{1}{d(k-1)}-\epsilon }\leq \Pi_{\max}^{\left(k\right)}\leq n^{\frac{1}{d(k-1)}+\epsilon }\right)=1.$$

**Remark 3.**  *We observe that the largest π-value in k-cluster persistence is significantly larger than that in i-dimensional homology. The main reason for this can be explained as follows. In [23], our upper bound for $\Pi_{i,\max}$ is an iso-perimetric inequality, which implies that large π-values require large connected components. However, the π-values in k-cluster persistence only require clusters of size k to be formed; thus, they can be generated by much smaller connected components.*

## Data Availability

No new data were created or analyzed in this study. Data sharing is not applicable to this article.

## Appendix A. Connectivity

**Proof of Theorem 2.** Note that for *k*-cluster filtration, connectivity is equivalent to the original G(n,p) graph having no components of size *j* for any k≤j≤n/2. Let $N_{j}=N_{j}\left(n,p\right)$ be the number of components of size *j* in G(n,p). Taking similar steps to the proof of connectivity for random graphs (e.g., [24]), we have

(A1) ENj≤njjj−2pj−1(1−p)j(n−j).

For k+1≤j<4k, we have

$$E\left\{N_{j}\right\}\leq Cn^{j}{\left(\frac{\Lambda }{n}\right)}^{j-1}e^{-j(n-4k)(\Lambda /n)},$$

for some C>0. Taking $\Lambda =\frac{1}{k}\left(\log n+\left(k-1\right)\log\log n\right)+c$, we have

$$E\left\{N_{j}\right\}\leq Cn^{-1/k}{\left(\log n\right)}^{j-1}e^{4j\log n/n}.$$

For 4k≤j≤n/2, we have

$$E\left\{N_{j}\right\}\leq {\left(\frac{ne}{j}\right)}^{j}j^{j-2}{\left(\frac{\Lambda }{n}\right)}^{j-1}e^{-j\Lambda /2}\leq \frac{n}{j^{2}}{\left(\frac{e^{1-c/2}\log n}{n^{1/2k}}\right)}^{j}\leq \frac{n}{j^{2}}n^{-j/3k}.$$

Therefore,

$$\sum_{j=4k}^{n/2}E\left\{N_{j}\right\}\leq \frac{1}{8k^{2}}n^{-1/3}.$$

To conclude, we show that

$$\lim_{n\to \infty }\sum_{j=k+1}^{n/2}E\left\{N_{j}\right\}=0.$$

This implies that for $\Lambda =\frac{1}{k}\left(\log n+\left(k-1\right)\log\log n\right)+c$, we have

$$P\left(G^{\left(k\right)}\left(n,p\right)\;\mathrm{is}\;\mathrm{connected}\right)\approx P\left(N_{k}>0\right).$$

Similar estimates to those above show that

$$E\left\{N_{k}\right\}\approx e^{-kc}.$$

Therefore, when c=w(n)→∞, we have $P(N_{k}>0)\to 0$. Together with a second-order argument, we can similarly show that when c=−w(n), we have $P\left(N_{k}>0\right)\to 1$. This concludes the proof.    □

**Proof of Proposition 1.** Recall that $N_{k}$ is the number of components of size *k* in G(n,r). In [25] (Theorem 3.3), it is shown that

$$E\left\{N_{k}\right\}\approx \mathrm{Var}\left(N_{k}\right)\approx C_{k}n\Lambda^{-(d-1)(k-1)}e^{-\Lambda },$$

for some constant $C_{k}>0$. When Λ=logn−(d−1)(k−1)loglogn+w(n), we have $E\left\{N_{k}\right\}\to 0$, implying that $P\left(N_{k}>0\right)\to 0$. When Λ=logn−(d−1)(k−1)loglogn−w(n), we can use Chebyshev’s inequality:

$$P\left(N_{k}=0\right)\leq P\left(|N_{k}-E\left\{N_{k}\right\}|\geq E\left\{N_{k}\right\}\right)\leq \frac{\mathrm{Var}\left(N_{k}\right)}{{\left(E\left\{N_{k}\right\}\right)}^{2}}\approx \frac{1}{E\left\{N_{k}\right\}}\to 0.$$

This completes the proof. □

## Appendix B. Maximal π-Value

**Proof.** Let r,R denote the birth and death radii of a cluster in a *k*-cluster persistence diagram and recall that π=R/r. For an upper bound, we denote the number of connected subsets of size *k* at radius *r* as $N_{k}\left(r\right)$. Using Mecke’s formula (cf. [26]),

(A2) $$\begin{array}{cc} E\left\{N_{k}\left(r\right)\right\} & =\frac{n^{k}}{k!}\int_{{\left(T^{d}\right)}^{k}}1\left\{G(x,r)\;\mathrm{is}\;\mathrm{connected}\right\}dx\\ \; & =\frac{n\lambda^{k-1}}{k!}\int_{{\left(R^{d}\right)}^{k-1}}1\left\{G((0,y),1)\;\mathrm{is}\;\mathrm{connected}\right\}dy,\\ \; & =C_{k}n\lambda^{k-1}, \end{array}$$

where $C_{k}$ is a positive constant, $\lambda =nr^{d}$, and we use the change in variables $x_{i}\to x_{1}+ry_{i}$ (i=2,…,k). For any ϵ>0, if $\lambda =n^{-1/(k-1)-\epsilon }$, then $E\left\{N_{k}\left(r\right)\right\}\to 0$. Thus, we can assume with high probability that the birth times of all *k*-clusters have $\lambda \geq n^{-1/(k-1)-\epsilon }$. In addition, from Theorem 1, if we denote $\Lambda =nR^{d}$, then when Λ=Clogn, the graph G(n,r) is connected. This implies with high probability that all death times of *k*-clusters have Λ≤Clogn. Together, these bounds imply with high probability that for all points in *k*-cluster persistence diagrams, for any ϵ>0, we have

$$\pi ={\left(\frac{\Lambda }{\lambda }\right)}^{1/d}\leq {\left(\frac{C\log n}{n^{-1/(k-1)-\epsilon }}\right)}^{1/d}.$$

Therefore, for any ϵ>0, we have

$$\lim_{n\to \infty }P\left(\Pi_{\max}^{\left(k\right)}\leq n^{\frac{1}{d(k-1)}+\epsilon }\right)=1.$$

For the lower bound, we denote the number of components of size *k* that are born before *r* and are isolated at radius *R* (and hence die after *R*) as ${\hat{N}}_{k}\left(r,R\right)$. Then,

$$\begin{array}{cc} E\{{\hat{N}}_{k}\left(r,R\right)\} & =\frac{n^{k}}{k!}\int_{{\left(T^{d}\right)}^{k}}1\left\{G(x,r)\;\mathrm{is}\;\mathrm{connected}\right\}e^{-n\mathrm{Vol}(B_{R}\left(x\right))}dx,\\ & =\frac{n\lambda^{k-1}}{k!}\int_{{\left(R^{d}\right)}^{k-1}}1\left\{G((0,y),1)\;\mathrm{is}\;\mathrm{connected}\right\}e^{-n\mathrm{Vol}(B_{R}\left(0,ry\right))}dy, \end{array}$$

where $B_{R}\left(x\right)$ is the union of balls of radius *R* around x. We apply the dominated convergence theorem, using the fact that when r/R→0, we have

$$\lim_{n\to \infty }\frac{\mathrm{Vol}(B_{R}\left(0,0+ry\right))}{\omega_{d}R^{d}}=1.$$

This leads to

$$E\{{\hat{N}}_{k}\left(r,R\right)\}\approx C_{k}n\lambda^{k-1}e^{-\omega_{d}\Lambda }.$$

Taking Λ=C>0 and $\lambda =n^{-1/(k-1)+\epsilon }$, we have

$$E\{{\hat{N}}_{k}\left(r,R\right)\}\to \infty .$$

Using a second moment argument shows that for all ϵ>0

$$P\left(\Pi_{\max}^{\left(k\right)}\geq n^{\frac{1}{d(k-1)}-\epsilon }\right)\to 1,$$

completing the proof.    □

## Appendix C. Limiting Persistence Diagram

The key part of the proof in [22] is bounding the add-one costs of the *persistent* Betti numbers. Let G=(V,E,W) be a weighted graph and let $\left\{G_{t}^{\left(k\right)}\right\}$ be the corresponding *k*-cluster filtration function. Define $\beta_{0}^{r,s}\left(G^{\left(k\right)}\right)$ as the 0-th persistent Betti number, i.e., the number of components born in t∈[0,r] that die at (s,∞] (for a formal definition, see [22]). Fix edge $e_{0}\notin E$ with a given weight $W\left(e_{0}\right)=w_{0}$ and let $\tilde{G}\;=\;\left(V,\tilde{E},\tilde{W}\right)$ be a weighted graph with $\tilde{E}=E\cup \left\{e_{0}\right\}$. Then,

$$\tilde{W}\left(e\right):=\left\{\begin{array}{cc} W(e) & e\neq e_{0},\\ w_{0} & e=e_{0}. \end{array}\right.$$

Let $\left\{{\tilde{G}}_{t}^{\left(k\right)}\right\}$ denote the corresponding *k*-cluster filtration function. The entire proof of Theorem 4 follows verbatim from the proofs in [22], provided that we prove the following lemma.

**Lemma A1.** 

$$\left|\beta_{0}^{r,s}\left({\tilde{G}}^{\left(k\right)}\right)-\beta_{0}^{r,s}\left(G^{\left(k\right)}\right)\right|\leq 1.$$

In other words, if we add a single edge to the filtration function, the number of persistent clusters can change by at most 1. Note that the proof here is not a straightforward application of Lemma 2.10 in [22] since in our case, when a single edge is added to the filtration function, the filtration values of other vertices and edges might be affected.

**Proof.** Let $e_{0}=\left(u,v\right)$ with $W\left(e_{0}\right)=w_{0}$. Let $C_{u}$ and $C_{v}$ denote the components of the end points of $e_{0}$ at $w_{0}$ in the original filtration function $\left\{G_{t}^{\left(k\right)}\right\}$. There are then three possible cases that can occur.

**Case I:** Both $|C_{u}|\;<k$ and $|C_{v}|\;<k$. Note that in this case, $\tau_{k}\left(u\right),\tau_{k}\left(v\right)>w_{0}$. Let $C_{u}^{\prime }$ be the cluster of *u* at $\tau_{k}\left(u\right)$, so that it is the component of *u* when it first appears in $G_{t}^{\left(k\right)}$. Define $C_{v}^{\prime }$ similarly. Note that aside from $C_{u}^{\prime }\cup C_{v}^{\prime }$, the filtration values of all other vertices remain unchanged by adding $e_{0}$.

Without loss of generality, suppose that $w_{0}<\tau_{k}\left(u\right)<\tau_{k}\left(v\right)$. Then, comparing the persistence diagrams for $G_{t}^{\left(k\right)}$ and ${\tilde{G}}_{t}^{\left(k\right)}$, only two differences can occur:

1. The point representing $C_{v}^{\prime }$ in $G_{t}^{\left(k\right)}$ is removed since $C_{v}^{\prime }$ is no longer a connected component in ${\tilde{G}}_{t}^{\left(k\right)}$ (as it is merged with $C_{u}^{\prime }$);
2. The point representing $C_{u}^{\prime }$ in $G_{t}^{\left(k\right)}$ may show an earlier birth time in ${\tilde{G}}_{t}^{\left(k\right)}$, within the interval $\left[w_{0},\tau_{k}\left(u\right)\right)$.

For a given r,s, the first change might decrease $\beta_{k}^{r,s}$ by 1, while the second change might increase it by 1. In any case, the total difference between $\beta_{0}^{r,s}\left({\tilde{G}}^{\left(k\right)}\right)$ and $\beta_{0}^{r,s}\left(G^{\left(k\right)}\right)$ is no more than 1.

**Case II:** $|C_{u}|\;\geq k$ and $|C_{v}|\;<k$. Defining $C_{v}^{\prime }$ in the same way as above, note that in this case, the filtration values of all points outside $C_{v}^{\prime }$ remain unchanged by adding $e_{0}$. The only change that occurs in this case is that the point in the diagram of $G_{t}^{\left(k\right)}$ corresponding to $C_{v}^{\prime }$ is removed in ${\tilde{G}}_{t}^{\left(k\right)}$ since it is now merged with $C_{u}$. Therefore, the difference in the persistent Betti numbers is at most 1.

**Case III:** Both $|C_{u}|\;\geq k$ and $|C_{v}|\;\geq k$. If $C_{u}=C_{v}$, then adding $e_{0}$ creates a 1-cycle (loop) and does not affect the *k*-cluster persistence diagram. If $C_{u}\neq C_{v}$, then both $C_{u}$ and $C_{v}$ are represented by different points in the persistence diagram of $G_{t}^{\left(k\right)}$. Adding $e_{0}$ causes one of these components to die earlier. In this case, $\beta_{0}^{r,s}$ may be decreased by 1 (if $s>w_{0}$).    □
